# Molecular subtyping of breast cancer intrinsic taxonomy with oligonucleotide microarray and NanoString nCounter

**DOI:** 10.1042/BSR20211428

**Published:** 2021-08-24

**Authors:** Yen-Jen Chen, Ching-Shui Huang, Nam-Nhut Phan, Tzu-Pin Lu, Chih-Yi Liu, Chi-Jung Huang, Jen-Hwey Chiu, Ling-Ming Tseng, Chi-Cheng Huang

**Affiliations:** 1 Comprehensive Breast Health Center, Department of Surgery, Taipei Veterans General Hospital, Taipei, Taiwan; 2 Division of General Surgery, Department of Surgery, Cathay General Hospital, Taipei, Taiwan; 3 School of Medicine, College of Medicine, Taipei Medical University, Taipei, Taiwan; 4 Bioinformatics Program, Taiwan International Graduate Program, Institute of Information Science, Academia Sinica, Taipei, Taiwan; 5 Graduate Institute of Biomedical Electronics and Bioinformatics, National Taiwan University, Taipei, Taiwan; 6 Institute of Epidemiology and Preventive Medicine, National Taiwan University, Taipei, Taiwan; 7 Department of Pathology, Cathay General Hospital SiJhih, New Taipei, Taiwan; 8 Department of Medical Research, Cathay General Hospital, Taipei, Taiwan; 9 Department of Biochemistry, National Defense Medical Center, Taipei, Taiwan; 10 Institute of Traditional Medicine, School of Medicine, National Yang Ming Chiao Tung University, Taipei, Taiwan; 11 School of Medicine, College of Medicine, National Yang Ming Chiao Tung University, Taipei Taiwan

**Keywords:** breast cancer, intrinsic subtype, molecular subtyping, NanoString nCounter, oligonucleotide microarray, PAM50

## Abstract

Breast cancer intrinsic subtypes have been identified based on the transcription of a predefined gene expression (GE) profiles and algorithm (prediction analysis of microarray 50 gene set, PAM50). The present study compared molecular subtyping with oligonucleotide microarray and NanoString nCounter assay. A total of 109 Taiwanese breast cancers (24 with adjacent normal breast tissues) were assayed with Affymetrix Human Genome U133 plus 2.0 microarrays and 144 were assayed with the NanoString nCounter while 64 patients were assayed for both platforms. Subtyping with the nearest centroid (single sample prediction (SSP)) was performed, and 16 out of 24 (67%) matched normal breasts were categorized as the normal breast-like subtype. For 64 breast cancers assayed for both platforms, 41 (65%, one unclassified by microarray) were predicted with an identical subtype, resulting in a fair κ statistic of 0.60. Taking nCounter subtyping as the gold standard, prediction accuracy was 43% (3/7), 81% (13/16), 25% (5/20), and 100% (20/20) for basal-like, human epidermal growth factor receptor II (HER2)-enriched, luminal A and luminal B subtypes predicted from microarray GE profiles. Microarray identified more luminal B cases from luminal A subtype predicted by nCounter. It is not uncommon to use microarray for breast cancer molecular subtyping for research. Our study showed that fundamental discrepancy existed between distinct GE assays, and cross-platform equivalence should be carefully appraised when molecular subtyping was conducted with oligonucleotide microarray.

## Introduction

Breast cancer is the most common female malignancy in Taiwan and ranks the fourth among cancer-related deaths according to the National Cancer Registry [1–3]. Despite increasing incidence, treatment outcomes of breast cancer are continuously improving with early detection through population-based screening and novel therapeutic agents [4,5]. Systemic therapy for breast cancer is determined from the presence or absence of estrogen receptor (ER), progesterone receptor (PR), and human epidermal growth factor receptor II (HER2) status as well as pathological factors such as nuclear grade and Ki-67 expression. Anatomy-defined prognostic factors for risk assessment include tumor size, axillary lymph node involvement and distant metastasis, constituting clinical or pathological [6].

On the other hand, scientists in molecular biology and clinical researchers had used microarrays to interrogate breast cancer as heterogeneous spectra based on gene expression (GE) profiles and distinct molecular subtypes were defined, with significant survival discrepancy observed [7–11]. Intrinsic subtypes, which were purposed by researchers from the Stanford University and University of North Carolina, constitute the major taxonomic classification for breast cancer since the past two decades. Initially Perou et al. identified 476 genes from 65 breast cancers and matched normal tissues. Four intrinsic subtypes were recognized through hierarchical clustering, namely basal-like, Erb-B2^+^, normal breast-like, and luminal epithelial/ER^+^ [8,9]. Subsequent update by Sorlie et al. further subdivided the luminal subtype into luminal A and B with luminal A breast cancers displaying the most optimistic prognosis in terms of distant metastasis [10]. Follow-up studies ascertained the wide existence of breast cancer intrinsic subtypes among diverse ethnic groups, including Taiwanese breast cancers with Han Chinese origin [11,12].

Intrinsic subtypes are determined from a pre-filtered gene set which vary the most across different subjects and vary the least within the same subject such as pre-/post-chemotherapy or primary/metastatic pairs [8]. The final version of intrinsic taxonomy, prediction analysis of microarray 50 gene set (PAM50) was trained from 189 breast cancers and 29 normal tissues and was validated from the combined dataset of 761 microarrays [13]. Constitutional 50 genes were the reduced gene set from the 1906 candidate genes of 189 breast cancers, of which 122 were profiled by both microarray and quantitative reverse-transcriptase polymerase chain reaction (qRT-PCR). Intrinsic subtypes defined by PAM50 was advocated as being prognostic and predictive independent of clinical factors such as ER, HER2, and proliferation markers.

The commercialized Prosigna breast cancer prognostic gene signature assay (Veracyte, Inc., South San Francisco, CA) is based on the PAM50 algorithm, and the nCounter platform (NanoString Technologies Inc., Seattle, WA) is adopted for both the academic PAM50 and commercial Prosigna [14]. Owing to the massive and rapidly accumulated public domain microarray depositories, it is quite intuitive to take advantage of these readily available GE data for molecular subtyping [15,16]. Robustness of subtyping results with oligonucleotide microarray, however, has rarely been addressed. The present study conducted PAM50 molecular subtyping for oligonucleotide microarray and NanoString nCounter, with some cases assayed for both platforms.

## Materials and methods

### Breast cancer samples recruitment

The whole study protocol (CGH-P101091) was approved by IRB of Cathay General Hospital. Enrolled subjects were those operated between 2010 and 2014. Written informed consent was obtained from all participants with full explanation by principal investigators (C.-C.H. and C.-S.H.) before surgery. Clinical data were retrieved from cancer registry; hormone receptor (HR) positivity was defined as at least 10% of nuclei with positive results of immunohistochemical (IHC) analysis of ER and/or PR while samples with low ER/PR phenotype (1–9% of nuclei with positive stains) were not enrolled. HER2 status was determined according to the ASCO and CAP guidelines; IHC 3+ and IHC 2+ with fluorescence *in-situ* hybridization (FISH) amplification indicated HER2 overexpression. Nuclear grade was determined based on the modified Bloom–Richardson (Nottingham) score.

### Nucleic acid extraction

Archived pathological slides and blocks of enrolled subjects were recruited from department of pathology. To extract nucleic acid form formalin-fixed paraffin-embedded (FFPE) tissues, paraffin was removed from specimens by xylene extraction then by ethanol washes. Sections with adjacent slides indicating more than 70% of cancer cells in surface area were selected, and total RNA was extracted by TRIzol reagent (Invitrogen, Carlsbad, CA) from 10-μm sections. RNA was purified with RNeasy mini kits (Qiagen, Germantown, MD) with integration checked by gel electrophoresis; samples with two bands of 18s and 28s passing quality check were prepared for downstream experiments.

### GE profiling with NanoString nCounter

RNA abundance was measured through digital multiplexed GE analysis using the NanoString nCounter system as previously described [17]. In brief, the collective CodeSet including target-specific reporters and capture probes were hybridized to regions of interest with covalently attached, target-specific sequences. Data import, quality control, and normalization of expression values were conducted with the nSolver version 4.0 (NanoString Technologies Inc.). Background subtraction from raw transcript counts was performed through negative input controls. Following housekeeping-normalization by dividing the geometric mean of six housekeeper-control genes (*ACTB*, *G6PD*, *RPLP0*, *TBP*, *TFRC*, and *UBB*), normalized counts were log2 transformed before further analyses.

### Microarray array experiments

GE analyses with oligonucleotide microarrays had been published [18]. In short, Affymetrix GeneChip Human Genome U133 plus 2.0 (Thermo Fisher Scientific, Waltham, MA) was adopted to profile messenger RNA extracted from freshly frozen cancerous or normal breast tissues obtained during surgery, and scanned images were processed with GeneChip Operating Software, Affymetrix’s Microarray Suite Software, and Transcriptome Analysis Console Software (Thermo Fisher Scientific) to generate detection *P*-values. For GE values, the robust multiarray average (RMA) algorithm was performed for perfect match probe signals [19]. When multiple probesets corresponding to a single gene, the one with the highest variability (defined by intersquadrant range) was selected.

### Single sample prediction

PAM50 analysis was performed using the published centroids on the normalized log2 GE data [13]. Fifty constitutional genes were mapped to the Affymetrix gene annotation file through the web-based NetAffx (www.affymetrix.com/analysis/index.affx). Breast cancers were assigned to one of the four or five intrinsic subtypes with the nearest centroid (single sample prediction, SSP) according to the method described previously [11,13]. For nCounter assay, the customized 145-gene NanoString Codeset BCeCSig already contained PAM50 genes.

Spearman’s rank correlation coefficients were used, and samples were designated as unclassified if correlation to all centroids were less than 0.1. To enhance comparability between the original discovering studies and independent breast cancer samples in current study, mean-centering of genes was performed, as suggested by the PAM50 investigators [20,21]. Gene-centering and normalization was performed independently within each platform. Additional systemic bias-adjustment, distance-weighted discrimination (DWD) was performed for microarray data with parameters set to ‘Standardized DWD’ for DWD type and ‘Center at the First Mean’ for mean adjust type. The first dataset, which had more samples and was considered as the standard one, was retrieved from Supplementary Tables S2 and S3 of Parker et al. for qRT-PCR data and from the Stanford Microarray Database for microarray data [13,22]. Missing values in prototypical GE data were imputed using K-Nearest Neighbor algorithm with default number of neighbors k = 10.

Downstream analyses were conducted with Genefu, an R/Bioconductor package, BRB Array-Tools version 4.6.1 (National Cancer Institute, Bethesda, MD) with built-in Prediction Analysis of Microarrays (PAM) algorithm and SAS version 9.4 (SAS Institute Inc., Cary, NC) for statistical analyses [23].

## Results

### Microarray study group

**Figure 1:**
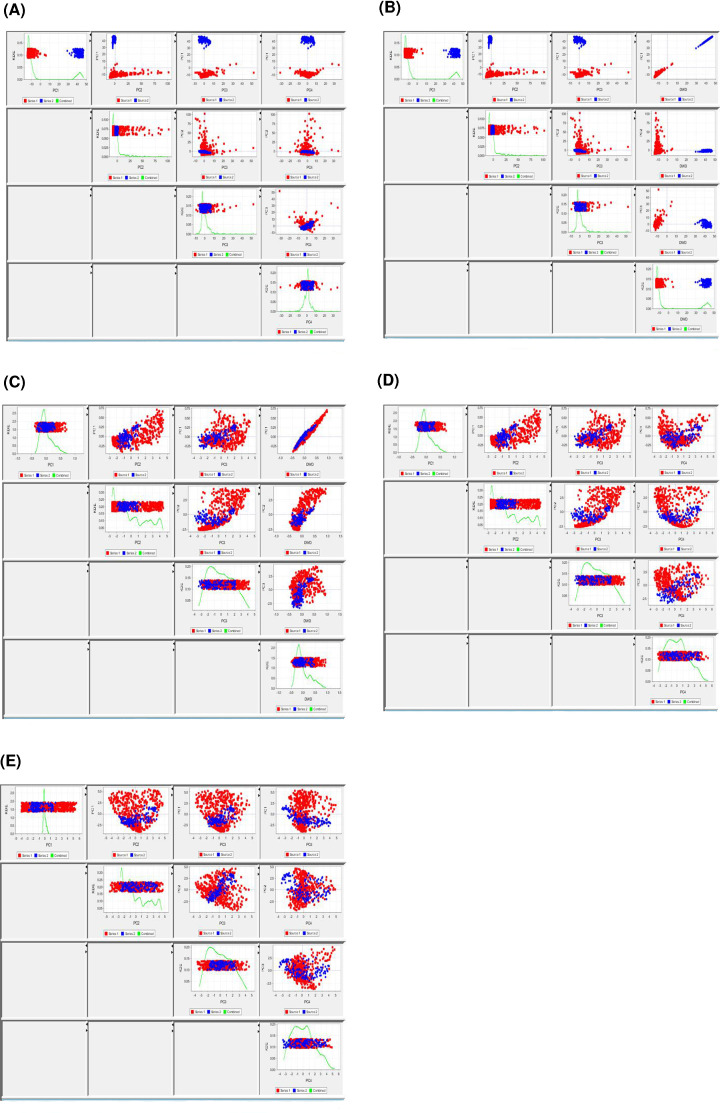
Visualization of diagnostics from DWD adjustment For raw data, PC directions are plotted in (**A**) while the first three PC directions with the fourth replaced by DWD vector in (**B**). For adjusted data, the first three PC directions of raw data with the fourth replaced by DWD vector are plotted in (**C**), PC directions of raw data in (**D**) and PC directions of adjusted data in (**E**).

A total of 109 breast cancers were successfully assayed with Affymetrix microarrays. Among them, 24 matched normal breasts were also profiled. Plots for visualization of diagnostics are detailed in Figure 1 for DWD adjustment.

Table 1 details PAM50 SSP tabulated with clinical IHC results. With five centroids used, 16 out of 24 (67.8%) normal breast samples were predicted as normal breast-like subtype. Among the remaining eight normal breast tissues not predicted as normal breast-like subtype, only one (12.5%) was predicted as the same luminal A subtype of matched cancer (Table 2).

**Table 1:** Distribution of IHC results and PAM50 intrinsic subtypes (without normal breast-like subtype) among 109 Taiwanese breast cancers assayed with oligonucleotide microarrays

IHC results	SSP				
	Basal-like	HER2-enriched	Luminal A	Luminal B	Total
HR+/HER2+	0	1	3	16	20
HR+/HER2−	2	1	13	35	52
HR−/HER2+	5	18	3	0	26
HR−/HER2−	7	1	3	0	11
Total	14	21	22	51	109^*^

* One HR+/HER2− case was unclassified by PAM50.

**Table 2:** Distribution of PAM50 intrinsic subtypes of 24 normal breast tissues (with normal breast-like subtype) and matched cancerous samples with oligonucleotide microarray

Matched cancerous samples	Normal breast tissues					
	Basal-like	HER2-enriched	Luminal A	Luminal B	Normal breast-like	Total
Basal-like	0	1	1	0	2	4
HER2-enriched	0	0	0	1	3	4
Luminal A	1	0	1	1	6	9
Luminal B	1	0	1	0	5	7
Total	2	1	3	2	16	24

Abbreviation: SSP2, SSP without normal breast-like subtype.

### nCounter study group

**Figure 2:**
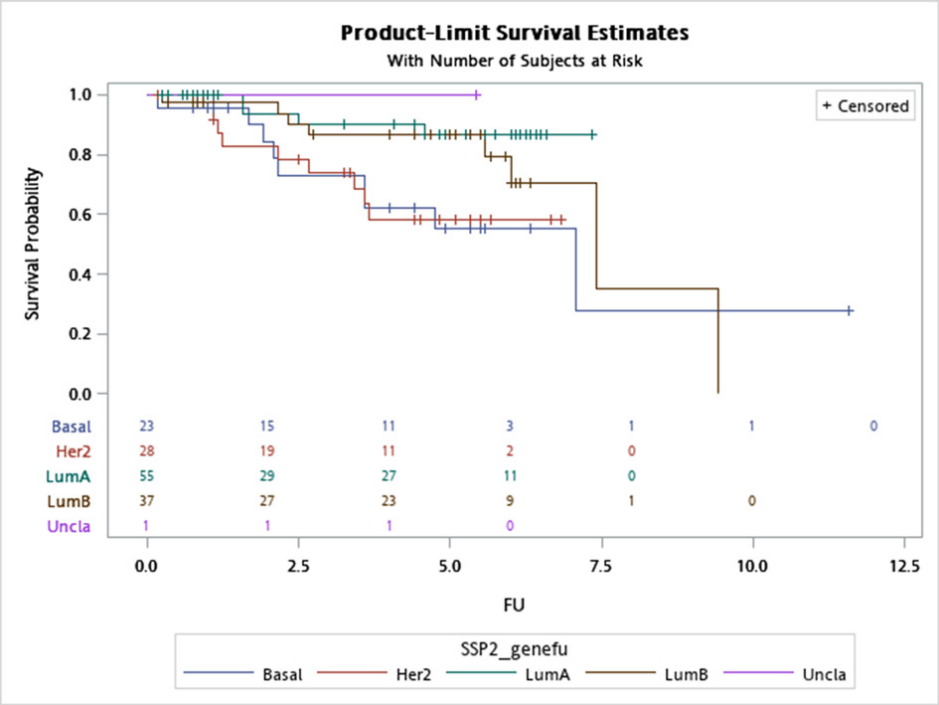
Overall survival stratified by PAM50 subtypes Overall survival among PAM50 SSP centroid-based luminal-A, luminal-B, basal-like, and HER2-enriched subtype (Log-rank test: 0.054, SSP2: PAM50 SSP without normal-like subtype, Basal: basal-like, HER2: HER2-enriched, Lum-A: luminal A, and Lum-B: luminal B subtype). During the 11.6 years of follow-up (median follow-up time: 5.3 years), there were 22 events (local recurrence, distant metastasis, or breast cancer-specific death) and 30 all-cause mortalities.

There were 144 Taiwanese breast cancers assayed with the NanoString nCounter platform, and Table 3 details the distributions of PAM50 SSP and IHC results (6 cases with missing IHC status). During the 11.6 years of follow-up (median follow-up time: 5.3 years), there were 22 events (local recurrence, distant metastasis, or breast cancer-specific death) and 30 all-cause mortalities. Figure 2 shows overall survival stratified by PAM50 subtypes (log-rank test: *P*=0.05). Percentages censored were 92.7, 78.4, 67.9, and 60.9% for luminal A, luminal B, HER2-enriched, and basal-like subtype, respectively.

**Table 3:** Distributions of IHC results and PAM50 intrinsic subtypes (without normal breast-like subtype) among 144 Taiwanese breast cancers assayed with the NanoString nCounter

IHC results	SSP				
	Basal-like	HER2-enriched	Luminal A	Luminal B	Total
HR+/HER2+	5	8	7	6	26
HR+/HER2−	4	2	41	30	77
HR−/HER2+	5	14	4	1	24
HR−/HER2−	7	3	0	0	11^*^
Missing	2	1	3	0	6
Total	23	28	55	37	144^†^

* One HR−/HER2− case was unclassified by PAM50.

† Six cases with missing IHC results.

### Comparison of subtype prediction between nCounter and oligonucleotide microarray

Because cross-platform comparison is one major interest of current study, breast cancers previously assayed with microarrays and with adequate archived tissues were preferentially recruited for NanoString nCounter experiments.

There were 64 breast cancers assayed for both platforms. Subtyping with the nearest centroid SPP was performed for each patient based on the transcriptional profiles from microarray and nCounter. Forty-one (65%) breast cancers were predicted with an identical subtype, resulting in a fair κ statistic of 0.60. Taking nCounter subtyping results as the gold standard, prediction accuracy was 43% (3/7), 81% (13/16), 25% (5/20), and 100% (20/20) for basal-like, HER2-enriched, luminal A and luminal B subtype based on oligonucleotide microarray GE profiles (Table 4). It deserved notice that one luminal A case predicted by nCounter was unclassified by microarray.

**Table 4:** PAM50 intrinsic subtypes prediction (without normal breast-like subtype) agreement between NanoString nCounter and oligonucleotide microarray

Microarray SSP2	nCounter SSP2				
	Basal-like	HER2-enriched	Luminal A	Luminal B	Total
Basal-like	3	0	0	0	3
HER2-enriched	1	13	1	0	15
Luminal A	0	3	5	0	8
Luminal B	3	0	14	20	37
Total	7	16	20^*^	20	64^*^

Abbreviation: SSP2, SSP without normal breast-like subtype.

* One luminal A case predicted by nCounter was unclassified by microarray.

## Discussion

With the high-throughput microarray technology, breast cancer is now regarded as a molecularly heterogeneous disease with wide transcriptional variations, and GE-based classifications have been developed such as the Amsterdam (70-gene) signature, Rotterdam (76-gene) signature, Genomic Grade Index and Recurrence Score (Oncotype DX), in addition to the Stanford/UNC intrinsic subtypes [25–28]. Most of these signatures are prognostic regarding relapse-free or distant metastasis-free survival [29]. Only the MammaPrint/BluePrint (the combination of the 70-gene and 80-gene signatures) as well as different generations of the intrinsic subtype signatures (Sørlie 500, Hu 306, and PAM50) are capable of molecular subtyping of breast cancers in a reproducible fashion [12,30–32].

Scientific and clinical values of breast cancer intrinsic taxonomy include information pertaining baseline prognosis, epidemiological discrepancy, and prediction to endocrine/targeted therapy. The optimistic long-term outcomes of luminal A breast cancers are evidenced by good prognostication inherited in this molecular subtype. Race and age difference in intrinsic subtypes have been reported as more basal-like subtype observed in African American population, resulting in a worse survival [33]. The predictive power of PAM50 risk classification comes from the synthetic ROR score, which incorporates correlation coefficients of four intrinsic subtypes with or without clinical tumor size and genomic proliferation score, estimating the benefit of endocrine therapy to balance the risk of sparing cytotoxic chemotherapy [34,35]. In addition, HER2-enriched subtype predicted response to neoadjuvant targeted therapy in HER2-positive breast cancers [36,37].

In current study we compared molecular classification of Taiwanese breast cancers profiled with both the NanoString nCounter and oligonucleotide microarray. The prediction consistency was evaluated with the κ statistic. Using microarray for molecular subtyping is frequently performed as the rapidly deposited public domain microarray datasets during the past two decades are readily available for scientific communities. Consequently, measuring invariance across distinct GE assessment platforms should be carefully appraised. Indeed, not all molecular subtyping was conducted with digital RNA counting of the nCounter system. For instance, the Cancer Genome Atlas Network (TCGA) had demonstrated that breast cancer intrinsic subtypes existed across five molecular levels including genomic DNA, DNA methylation, exome sequencing, messenger RNA and micro RNA [38]. In this study, RNA sequencing (RNA-Seq) and Agilent microarray was the platform for molecular subtyping and independent studies also ascertained the correlation (Spearman’s correlation coefficients to the nearest centroid > 0.89) and concordance (96%) between RNA-Seq and digital multiplexed GE in 96 triple negative breast cancers [39].

The agreement of the centroid-based PAM50 SSP between the nCounter and microarray was only substantial to fair, and only 41 out of the 63 samples (65%, one unclassied) showed an identical subtype, resulting in a κ statistic of 0.60. Luminal A subtype is the least concordant category (25% agreement), followed by basal-like (43%), HER2-enriched (81%), and luminal-B (100%) subtype. In other words, microarray identified more luminal B from luminal A subtype predicted by nCounter, and this differential shift compromised prediction consistency.

Our experiments showed that fundamental discrepancy existed between distinct approaches for GE measurement. Compared with the dual probes design (50-mer each) of the nCounter target-binding domain, the relatively short 25-mer design of the Affymetrix oligonucleotide microarray as well as the summative probe set GE value (20 perfect match/mismatch probe pairs designed to assay the transcript of a single gene) inevitably introduces baseline measurement variation. In addition to fundamental discrepancy in biochemistry, whether the processes of background correction, global/housekeeping genes normalization, outlier detection were performed or not, as well as the identities of housekeeping genes, wound impact molecular subtyping analyses enormously. Probe affinity and non-overlapping targeted regions of investigated genes might result in a non-linear relationship between transcriptional profiles measured from distinct GE assays and compromised subtype prediction consistency. Delmonico et al. also conducted side by side comparisons between the Affymetrix microarray and NanoString nCounter, and concluded that although correlation among samples within the same platform was quite high (range from 0.90 to 0.99), the correlation among samples between the two different platforms was poor (range from 0.4 to 0.5, [40]).

For GE subtype assignment, another variable that may influence the results other than technological platform and prediction algorithm, is the subtype composition of assayed samples. Since the dataset needs to be normalized and gene-centered before applying PAM50 classifier, the method is sensitive to the subtype composition of the cohort [41]. In current study, gene-centering and normalization was performed independently within each platform: i.e. 133 microarrays and 144 nCounter samples were processed separately. DWD adjustment was further carried out for microarray data and Figure 1 shows diagnostic visualization eliminating systemic bias between platforms.

Taking nCounter-defined molecular subtypes as the gold standard, treatment outcomes varied enormously (Figure 2). Luminal A breast cancers enjoyed a more than 90% of overall survival at the end of follow-up (30 November 2019), which was in agreement with our previous studies [12,24,41]. Pertaining IHC phenotypes, only 7 out of 23 (33.3%, two with missing IHC results) basal-like breast cancers were clinically triple negative (HR−/HER2−), while for 28 HER2-enriched breast cancers, 22 (81.5%, one with missing IHC result) were clinically HER2+. Among 55 luminal A subtype breast cancers, 41 (78.8%, three with missing IHC results) were IHC HR+/HER2− and only 4 luminal A breast cancer was clinically HR negative. Finally, there were 30 (83.3%) HR+/HER2− and 6 (16.7%) HR+/HER2+ breast cancers within 37 luminal B population (one missing IHC). Lack of perfect concordance between molecular subtypes and IHC phenotypes has been known for a long time since the introduction of PAM50 taxonomy, and our results highlighted the necessity of the same digital RNA counting workflow to designate unbiased and reproducible intrinsic subtypes [13,42,43].

The present study demonstrated the fundamental discrepancy existed between microarray and nCounter measured transcriptional profiles, and more caution should be placed pertaining measuring equivalence without too much extrapolation. On the other hand, less than half of adjacent normal breast tissues were predicted as the normal breast-like subtype and for those not predicted as normal breast-like subtype, around thirty percent displayed similarity with corresponding cancer samples (all luminal A subtype). This finding is in agreement with the declaration of Parker et al. as they neglected normal breast-like subtype in clinical use since the introduction of PAM50 and regarded this subtype as normal breast tissue contamination [13].

There were some limitations of the study. First, microarray and nCounter experiments were not performed synchronously. Oligonucleotide microarrays were conducted for prospectively enrolled freshly frozen cancerous and matched normal breasts while for NanoString nCounter, retrospectively retrieved pathological archives were profiled. The nCounter assay was designed to be run on paraffin sections and only the tumor-rich area of the slide was used to ensure high tumor content. On the other hand, freshly frozen tissue was the input for microarray experiments. The inherited difference in tumor composition between FFPE and freshly frozen samples, tumor heterogeneity and spatial differences in sampling site might influence subtyping results substantially. Second, housekeeping genes adopted in current study were not exact as those selected by the original investigators of PAM50 (*ACTB*, *GUSB*, *MRPL19*, *PSMC4*, *PUM1*, *RPLP0*, *SF3A1*, and *TFRC*) and only three genes (*ACTB*, *RPLP0*, *TFRC*) were common. The discrepancy in housekeeping genes might introduce some comparability bias. In upcoming version 2 of BCeCsig CodeSet we will evaluate the impact of housekeeping genes selection upon subtyping results. Third, case number of both cohorts, and well as the intersect of 64 breast cancers, was only modest, which might impede external validation of the study [44]. Finally, proliferative markers, which also constitute the Prosigna score, were not interrogated in current study, and prognostic relevance of intrinsic subtypes was not fully addressed. However, the differential shift from luminal A to luminal B subtype might overestimate breast cancer risk when microarray was used for molecular subtyping.

## Supplementary Material

Supplementary Tables S1-S3

## Data Availability

Part of microarray experiments (*n*=81) had been deposited at the NCBI Gene Expression Omnibus (GEO) under the accession numbers GSE48391 and GSE146558 (*n*=109) [18]. A new GEO accession number GSE162228 containing the 24 microarrays of normal breast tissues will be available once the manuscript is accepted. The full data of current study are available in Supplementary Tables S1 and S2. Supplementary Tables S3A,B detail demographic features of breast cancers assayed for oligonucleotide microarray and NanoString nCounter.

## Abbreviations

DWD: distance-weighted discrimination
ER: estrogen receptor
FFPE: formalin-fixed paraffin-embedded
GE: gene expression
GEO: Gene Expression Omnibus
HER2: human epidermal growth factor receptor II
HR: hormone receptor
IHC: immunohistochemical
PAM50: prediction analysis of microarray 50 gene set
PR: progesterone receptor
qRT-PCR: quantitative reverse-transcriptase polymerase chain reaction
RMA: robust multiarray average
RNA-Seq: RNA sequencing
SSP: single sample prediction
